# Data concerns undermine AI applications for climate

**DOI:** 10.1038/s44168-026-00390-2

**Published:** 2026-06-06

**Authors:** Emilie Vrain, Charlie Wilson

**Affiliations:** 1 Environmental Change Institute, University of Oxford, Oxford, UK; 2 International Institute for Applied Systems Analysis (IIASA), Laxenburg, Vienna, Austria

**Keywords:** Environmental social sciences, Climate-change mitigation

## Abstract

The global climate crisis demands innovative solutions, and artificial intelligence (AI), including large language models (LLMs), offers significant potential to improve efficiency and enable lower-carbon lifestyles. However, public concerns around data privacy may affect the use of AI-enabled, data-driven applications with potential climate benefits. Drawing on three empirical studies with a nationally representative UK sample (*N* = 2078), we show that heightened awareness of AI’s data collection practices reduces willingness to engage with such technologies - especially when perceived user benefits are low. We also find that data protection behaviours and greater familiarity with AI are associated with lower perceived risk, suggesting adaptive responses rather than disengagement. These behavioural dynamics highlight the importance of transparency, user agency and the integration of human and environmental dimensions into existing AI governance frameworks. Our results illustrate how public perceptions and everyday practices influence the social acceptance of digital innovations for sustainability.

## Introduction

Recent discussions on artificial intelligence (AI) and climate change emphasise AI’s dual potential for substantial positive and negative impacts. On the one hand, AI has been identified as a potential tool for reducing greenhouse gas emissions^1–4^. For example, a recent review estimates that targeted AI applications in power, food and light-vehicle transport could reduce global emissions by 3.2–5.4 GtCO_2_e per year by 2035 under optimistic deployment scenarios^4^. On the other hand, the latest wave of development in AI capabilities and applications, particularly through large language models (LLMs) and generative systems, has expanded computing demands. These advances enable new adaptive and creative capabilities but also lead to soaring energy use in data centres worldwide^5–7^, raising concerns over the sector’s growing carbon footprint^4,8^.

Beyond these direct impacts lie subtler yet increasingly influential dimensions of AI’s climate impacts: the ways in which AI reshapes consumer engagement and aggregate consumption patterns. AI applications in areas like micro-target marketing and agentic systems could indirectly influence societal energy demands, potentially increasing consumption^9,10^. At the same time, the same underlying technologies can support lower-carbon lifestyles through a range of digital tools that encourage more sustainable choices.

We use the term AI to denote data-driven, learning-based systems that infer from inputs to generate predictions or decisions^11^, encompassing both machine-learning models and newer generative approaches that extend earlier forms of digitalisation^3^. This framing is relevant to the present study as many data-driven business models increasingly integrate AI functionalities across everyday consumer domains (see Supplementary Table 1). Examples include smart energy management tools that use machine learning to learn household routines and optimise energy use; peer-to-peer (P2P) platforms in retail and mobility that employ predictive analytics to manage collaborative consumption through usership business models or more efficient resource use^12–14^. These AI-enabled, climate-relevant applications have the potential to contribute meaningful carbon reductions by changing daily behaviours, yet their benefits depend on how they are adopted and used^9^. Their impact may be positive by replacing high-carbon behaviours, or negative if they lead to increased usage and energy consumption through rebound effects^15,16^.

Despite the growing landscape of climate-relevant applications, a critical knowledge gap remains: how public concerns about AI, particularly around personal data use, affect the use and impact of these technologies. Prior research has examined drivers of sustainable technology use (e.g.^17^) and data privacy concerns in digital tools^18–20^ but not their intersection in the context of climate-relevant applications. This oversight is increasingly consequential as AI becomes more utilised and more salient in public discourse and the demand for climate action accelerates. This study addresses the gap by investigating how data privacy concerns, amplified by perceptions of AI risk, influence use of climate-relevant applications, and what factors alleviate or exacerbate these effects.

The urgency of this investigation is further highlighted by the peak in public attention to AI during 2023-24, following the launch of ChatGPT, which our study directly exploits, reflecting on changes in perceptions over the year. With generative AI (GenAI) tools like ChatGPT becoming ‘the fastest application to reach 100 million users’^21^, AI has surged in prominence with considerable media attention, raising the salience of its usage across sectors^22–24^. Public surveys report heightened perceptions of AI risk, distrust, and concerns over opaque data use^25–29^, amplified by high-profile data misuse scandals (e.g. Cambridge Analytica’s misuse of Facebook data^30^ and Clearview AI’s image-scraping^31^).

AI governance has progressed including through the EU’s AI Act (Regulation (EU) 2024/1689), however, risk mitigation focuses on system safety, fundamental rights and trustworthiness. There remain notable gaps not only in the need to align AI systems with sustainability^32–34^ but also to account for how public trust, privacy concerns and perceptions of AI risk affect societal uptake of tools that could support climate goals.

As AI technologies become more prominent in society, we argue that public awareness of data practices increases in tandem, shaping perceptions of trust and influencing the use of AI-enabled tools which have potential climate benefits. Are data concerns undermining AI’s contribution to tackling climate change? Although we reason that data privacy concerns play a key role, it is overly simplistic to view them as the sole determinant of technology use. A range of other psychological factors—including perceived ease of use, perceived functionality, and environmental motivation—are known to influence both usage and usage propensity^35,36^. We explore how the salience of AI’s data collection practices interacts with these other drivers of technology acceptance.

While AI applications are highly visible in domains like targeted advertising —where personalisation is overt and widely discussed^37^, their use in shared mobility or food waste reduction platforms tends to be less explicitly communicated such that users may be unaware of their data-driven functionalities. The concept of the privacy paradox describes how individuals express concerns about data privacy yet continue to engage in behaviours that expose their personal data^38,39^. Although debated for oversimplifying the relationship (e.g.^40,41^), we use it here as a heuristic framework to examine how users navigate tensions between privacy concerns and technology use. Two explanations commonly offered for the paradox are: (1) individuals weigh the perceived benefits of using a technology against its abstract privacy risks (so-called privacy calculus) and (2) decisions are made in the absence of clear awareness about how data are collected, used, or shared^41^. We argue that while privacy concerns may deter some users, others engage in protective behaviours that help manage perceived risks and sustain engagement with applications.

**Fig. 1:**
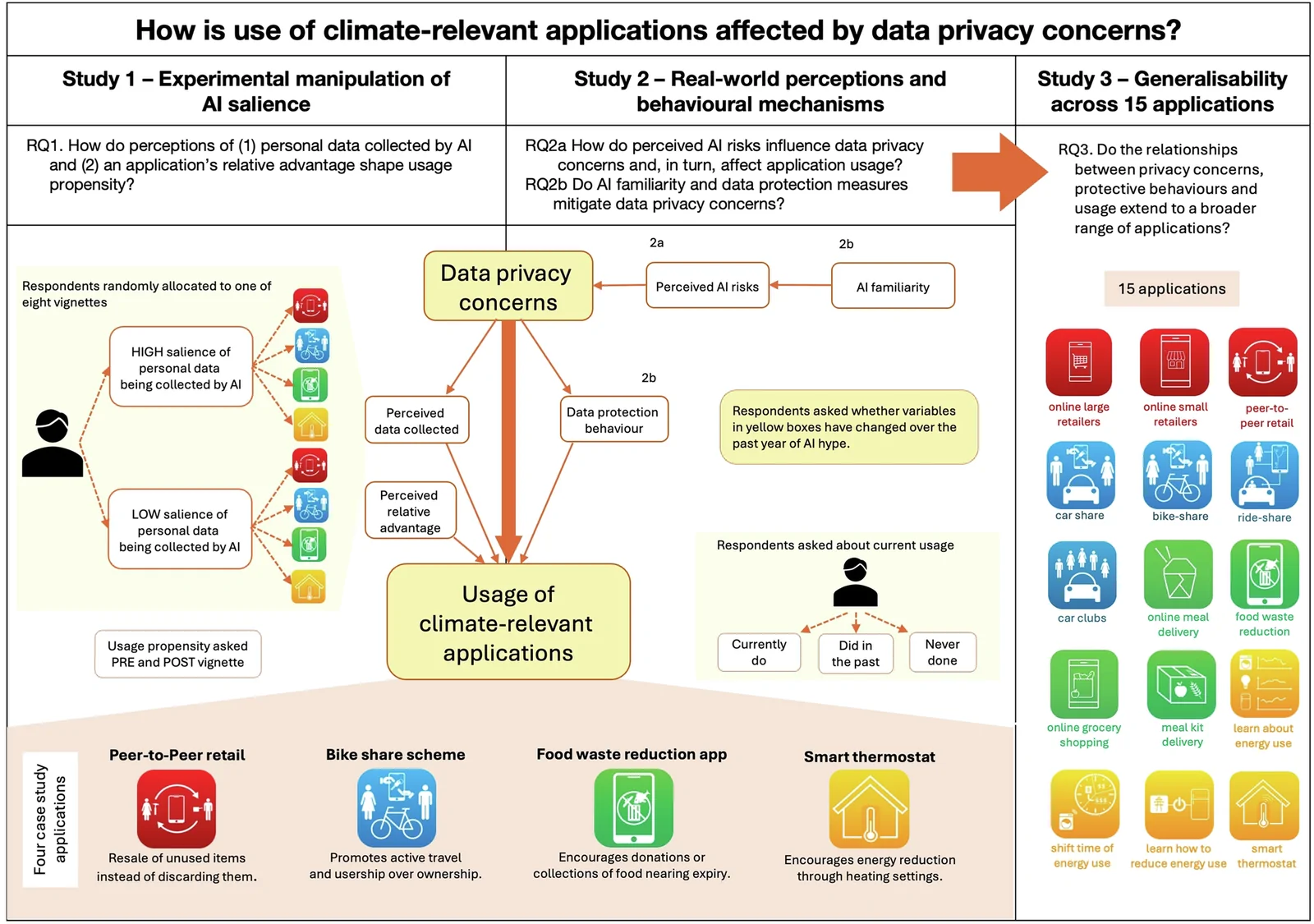
Summary of the research design framework. Study 1: Respondents were randomly assigned to one of eight vignettes describing a scenario of using a climate-relevant application, emphasising either (1) high salience or (2) low salience of AI collecting personal data for functionality. Respondents were asked about relative advantage, perceived data collection and usage propensity. Study 2: Respondents were asked about their familiarity of AI (including exposure to information about AI and its importance, ability to recognise AI, seeking information about AI, and use of GenAI), perceived AI risks, current data privacy concerns, data protection behaviours, application usage frequency, usage propensity and whether some of these have changed over the previous year of AI hype. Study 3: Respondents were asked about their application usage frequency for an additional 11 climate-relevant applications across daily life domains.

In this paper, we offer a novel, empirically grounded investigation into how the use of climate-relevant applications is affected by data privacy concerns and what factors influence these effects. We do so through three studies using a nationally representative UK sample (*N* = 2,78). Figure 1 provides an overview of the research design. Study 1 and 2 examine four case study climate-relevant data-driven applications across domains of daily life: (1) retail – P2P retail platform; (2) mobility – bike share scheme platform; (3) food – food waste reduction app; (4) home - smart thermostat. Although these applications are not AI systems in a technical sense, each incorporates AI or machine-learning functionalities that underpin personalisation, prediction, or optimisation, making them relevant to understanding how users perceive AI’s role in everyday digital services.

Study 1 uses a vignette experiment to test how varying levels of AI data collection salience affect perceived data collection and usage propensity. The impact of perceived relative advantage is also explored. Study 2 investigates how perceived AI risks influence data privacy concerns, and in turn how concerns impact the usage of the four applications. It also examines how changes in privacy concerns over the past year relate to application usage. Additionally, we study whether the recent surge of information and awareness of GenAI— often termed the ‘GenAI hype’^42–44^—influences perceived AI risks, as well as whether behaviours around data protection positively mediate privacy concern’s impact on application use. Finally, Study 3 tests the generalisability of our findings across a diverse set of 15 climate-relevant applications.

## Results

### Study 1 Impacts of AI data collection practices and relative advantage

The salience of AI collecting personal data significantly reduced the *usage propensity* for climate-relevant applications (mean reduction −0.260, 95% CI [−0.350 to −0.170], *p* < 0.001, *r* = −0.161 small effect). This effect was particularly pronounced for P2P retail platforms and smart thermostats, shifting respondents’ usage propensity from ‘likely’ to ‘unlikely’. Even low salience of AI collecting data reduced propensity for P2P retail platform usage, suggesting inherent concerns regarding data practices in such applications. No significant effects were observed for food waste reduction apps, whereas salience increased *usage propensity* for bike share schemes; however, respondents remained ‘unlikely’ to use them (Supplementary Table 2).

To assess whether our vignette experimental design effectively manipulated perceptions of AI collecting personal data [*perceived data collected*], we examined differences in responses to the question *‘When using this application, it would collect personal data about me.’* Responses were captured on a five-point Likert scale following presentation of each vignette. Participants exposed to a high AI salience vignette were more likely to perceive that personal data was being collected compared to those exposed to a low AI salience vignette (*U* = 606167.50, *z* = 5.169, *p* < 0.001, *r* = 0.113 small effect). Differences in perceptions were significant for P2P retail platforms (*U* = 35082.50, *z* = 2.946, *p* = 0.003, *r* = 0.132 small effect) and bike share schemes (*U* = 40931.00, *z* = 4.475, *p* < 0.001, *r* = 0.196 small effect) but not for other applications (Supplementary Table 3). While our vignette-based experimental design effectively introduced variation in perceptions of AI data collection practices, the resulting effects differed across applications. Because our main interest lies in the role of perceived data collection, regardless of vignette assignment, we shift our analysis from between-vignette comparisons to participants’ responses on *perceived data collection*. This enables us to better capture the underlying causal and mediating mechanisms in our ex-post framework.

Structural equation modelling (SEM) results indicate that the association between *data privacy concerns* and *usage propensity* is fully mediated by *perceived data collected*, ultimately reducing *usage propensity* across all four climate-relevant applications (Table 1, with model fit indices provided in Supplementary Table 4). *Data privacy concerns* have a positive effect on *perceived data collected*, and *perceived data collected* has a negative effect on *usage propensity*. These findings suggest that concerns about data privacy shape individuals’ willingness to use AI applications by influencing their perceptions of AI data collection practices. Thus, perceptions of such practices potentially act as a psychological barrier, amplifying the impact of privacy concerns on usage decisions.

**Table 1:** Study 1’s test for mediation using a Bootstrap analysis with a 95% Confidence Interval

Study —Path analysis	Data privacy concerns → P*erceived data collected* → Usage propensity								Perceived relative advantage → Usage propensity	
		Direct effect	*P*	Indirect effect	95% CI		*P* (2-tailed)	Mediation conclusion	Direct effect	*P* (2-tailed)
	*n*	*β*	(2-tailed)	*a* × *b* = *β*	Low	High			β	
P2P retail platform	476	−0.005	n.s.	-0.005	-0.010	-0.001	0.001	full	1.255	<0.001
Bike share scheme	513	−0.010	n.s.	-0.004	-0.010	-0.001	0.002	full	0.748	<0.001
Food waste reduction	512	−0.003	n.s.	-0.009	-0.016	-0.004	<0.001	full	1.086	<0.001
Smart thermostat	519	0.010	n.s.	-0.003	-0.007	0.000	0.021	full	1.014	<0.001

Unstandardised coefficients reported. Bootstrap Sample = 5000 with replacement.

aEffect of the independent variable on the mediator.

bEffect of the mediator on the dependent variable.

**Fig. 2:**
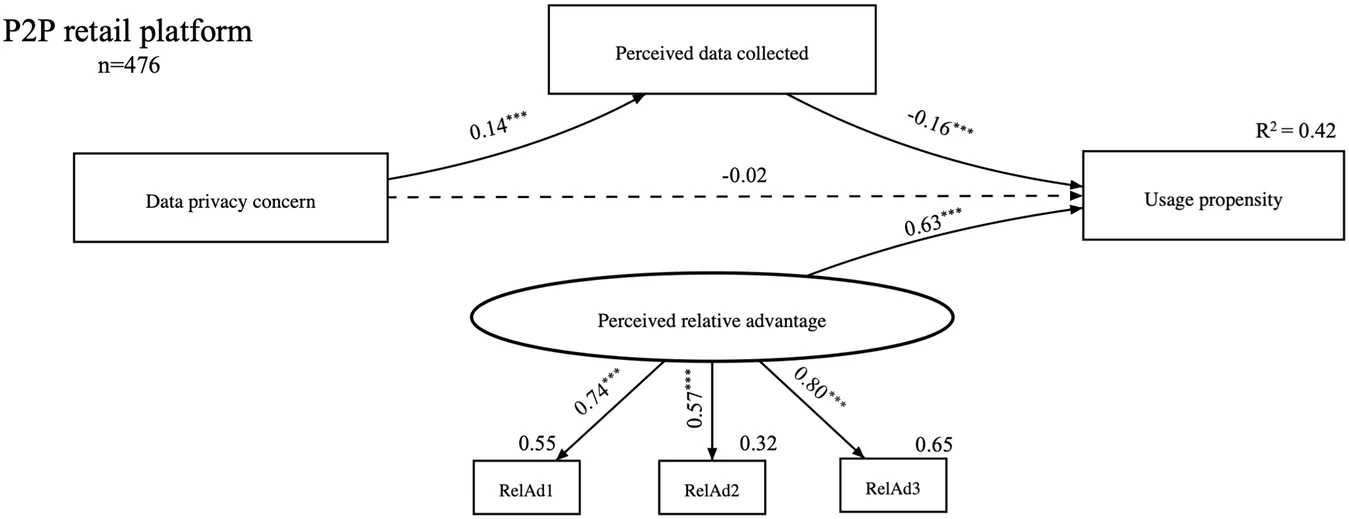
Structural equation model showing the direct and indirect effects of data privacy concern, perceived data collected and relative advantage on usage propensity of a P2P retail platform (measured post-vignette). The latent variable ‘perceived relative advantage’ consists of: RelAd1 ‘better than alternatives’, RelAd2 ‘easy to use’ and RelAd3 ‘useful’. Numbers adjacent to the arrows are standardised path coefficients and indicative of the effect size of the relationship. Dashed lines indicate nonsignificant relationships. *P < 0.05, **P < 0.01 and ***P < 0.001. *R*^2^ represents the proportion of variance explained by the relations in the path model.

We next demonstrate that *perceived relative advantage* acts as a counterbalancing influence on users’ concerns over data privacy (e.g. for P2P retail *λ* = 0.63, *p* < 0.001; Fig.2). This is consistent with privacy calculus theory which characterises how digital users trade off benefits with privacy risks. We find *perceived relative advantage* has the strongest effect on *usage propensity* for P2P retail platforms and smart thermostats (Supplementary Fig. 1).

We have shown that AI salience amplifies data privacy concerns that undermine usage propensity of climate-relevant applications, particularly if their relative advantage is weak. However, this was through an experimental manipulation of AI data collection salience. In our second study, we test the validity of our findings given current levels of AI awareness particularly over the past year during the GenAI hype.

### Study 2 Impacts of perceived AI risks, familiarity and data protection behaviours

Existing perceptions of AI are strongly biased towards AI-related risks: 36% of Study 2 participants think AI poses greater risks than benefits – double the proportion who view its benefits as outweighing its risks (18%) (Personal characteristics of respondents across three risk-benefit subgroups are summarised in Supplementary Table 5). The ONS-derived question did not specify a type of risk ^25^, but its placement after the privacy and data-protection blocks likely primed respondents to think of risks relating to data misuse. The most frequently cited perceived negative impact of AI was *‘use of personal data without consent,’* reported by 72% of participants. These concerns are consistent with population-level trends^25^. *Data privacy concerns* increased over the past year, while *usage* of the four climate-relevant applications decreased (Supplementary Table 6). Together, these findings highlight potential widespread issues about AI’s implications for data privacy and trust.

*Perceived AI risks* were positively associated with self-reported *data privacy concerns* (*r*_*s*_ = 0.163, *p* < 0.001) and *changes in concerns* over the past year (*r*_*s*_ = 0.201, *p* < 0.001) (Supplementary Table 7a). However, although high privacy concerns are expected to deter application usage, we find no significant difference in levels of concern between users and non-users of climate-relevant applications (Supplementary Table 8). We also find significant positive correlation between *changes in data privacy concerns* and *changes in application usage* (Supplementary Table 7b). These findings on real-world behaviour are consistent with the privacy paradox that suggests why users report concerns yet continue using applications. Can data protective behaviours and familiarity with AI help further counter the undermining effect of AI risks and data privacy concerns on use?

First, AI familiarity through *exposure to AI-related information* and the *perceived importance of information source* (e.g. media, social interactions) were associated with lower *perceived AI risks*, though effect sizes were weak (Cramer’s V < 0.2 for exposure and Spearman’s rho values ≈ 0 for importance - Supplementary Table 9 and Table 7c). Stronger associations emerged for other aspects of AI familiarity, namely proactive interactions, where *actively seeking AI-related information*, *recognising AI use*, or *using ChatGPT* correlated with lower *perceived AI risks* (*r*_*s*_ = −0.211, 0.219, 0.317, *p* < 0.001, Supplementary Table 7d). Results suggest that such familiarity may habituate use and potentially help reduce uncertainty around AI risks.

Second, general *data-protection* behaviours to safeguard personal information online (e.g. removing location tracking, creating a difficult-to-guess/strong password) fully mediated the relationship between *data-privacy concerns* and *usage frequency* for P2P retail platforms, food-waste reduction apps and smart thermostats. For bike share schemes, partial mediation was found as *data privacy concerns* were significantly negatively associated with *usage frequency* (*λ* = −0.010, *p* = 0.007) (Supplementary Tables 10 and 11). These behaviours are not AI-specific but apply broadly across data-driven digital environments, which underpin the functioning of AI-enabled applications.

We show that proactive engagement with AI, especially usage of GenAI, may help reduce perceived AI risks, and that data protection behaviours possibly help alleviate the potential negative impact that data privacy concerns have on the usage of four climate-relevant applications. Do these findings hold for a more diverse set of data-driven climate-relevant applications? In Study 3 we expand our investigation to an additional 11 applications and explore data privacy concerns and the mediating role of data protection behaviours on usage.

### Study 3 Impacts on a broad range of climate-relevant applications

**Fig. 3:**
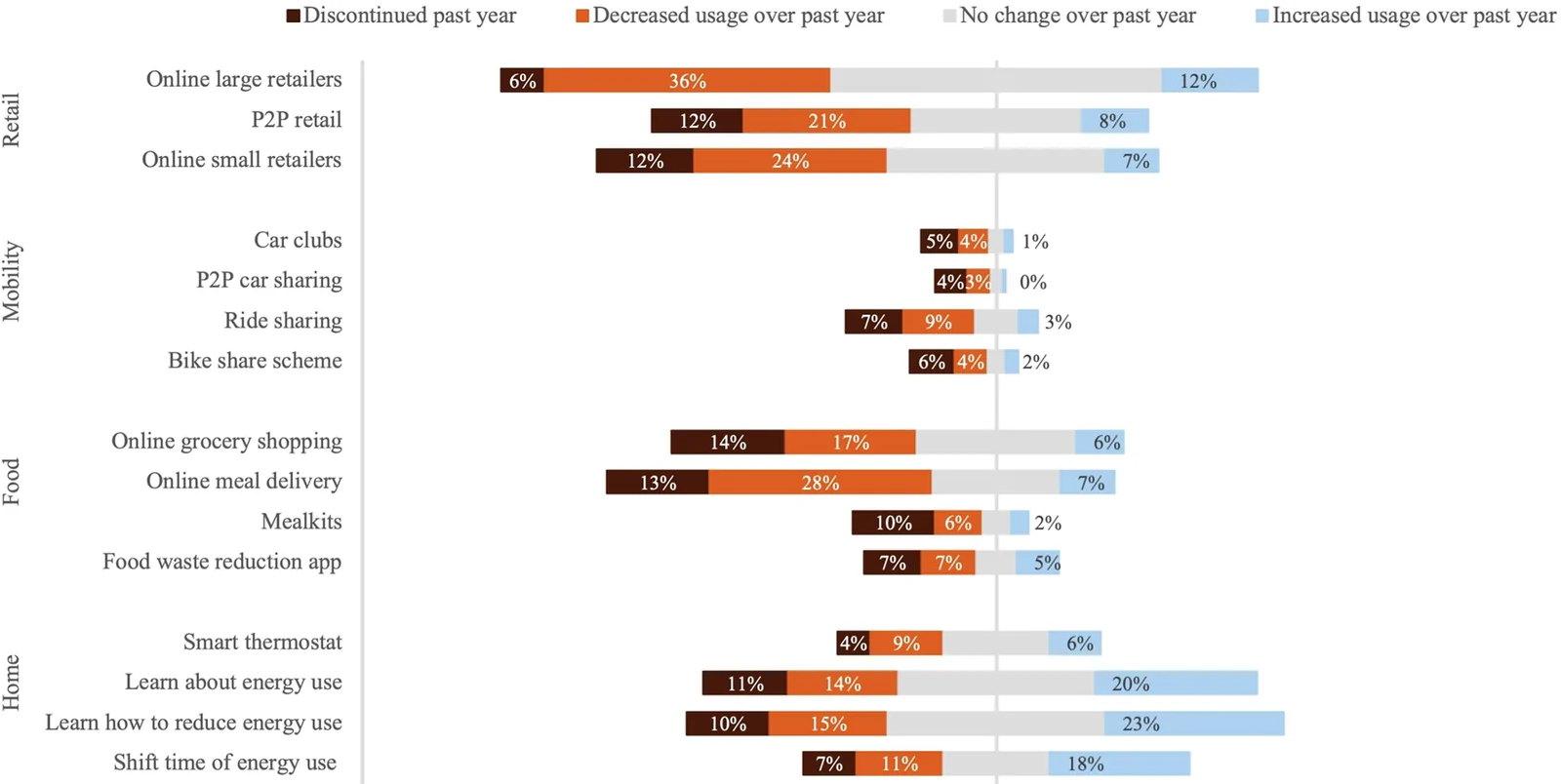
Self-reported change in usage of 15 climate-relevant applications over the past year. The percentage of participants (*n* = 2078) reporting changes in their use of 15 data-driven applications compared to one year prior to the survey. Applications are grouped into four domains: retail, mobility, food, and home energy. For each application, respondents indicated whether their usage had discontinued, decreased, remained unchanged, or increased over the past year. Horizontal stacked bars show the distribution of responses. Dark brown bars represent discontinued use, medium brown bars represent decreased usage, light grey bars represent no change in usage, and light blue bars represent increased usage. Percentages are shown within each segment; totals may not equal 100% due to rounding. The figure presents descriptive proportions only.

Broadening the investigation to a wider range of data-driven climate-relevant applications, trends show a decline in usage over the past year across home, food, mobility and retail domains (Fig. 3). SEMs revealed that *data privacy concerns* do not uniformly deter usage; instead, their effect is largely mediated through *data protection* behaviours, which in many cases are associated with higher *usage frequency*. For most applications, *data privacy concerns* were fully mediated, suggesting that rather than disengaging, concerned individuals adopt strategies of data protection and continue use (Supplementary Table 12). Privacy concerns may motivate individuals to implement protective strategies, allowing them to navigate data privacy risks without forgoing an application’s benefits, a novel finding to contribute to discussions on the privacy paradox.

Notably, for bike share schemes (reported in Study 2) and meal kits, *data privacy concerns* retained a significant negative direct association with *usage frequency* (meal kits *λ* = −0.009, *p* = 0.048), even after accounting for *data protection* behaviours. In contrast, informational energy applications – particularly those aimed at helping users reduce energy consumption i.e. ‘learning how to reduce energy use’, exhibited a small but significant positive direct association between *data privacy concerns* on *usage frequency* (*λ* = 0.025, *p* < 0.001) (Supplementary Table 12). This finding diverges from the broader trend, suggesting that in contexts where AI is perceived as non-invasive and empowering for users, the benefits (e.g. gaining energy-saving knowledge) outweigh the minimal privacy risks.

Finally, it is important to note that model fit was stronger for retail and home energy applications, suggesting greater clarity or salience of data-driven functionalities in these domains (Supplementary Table 13).

## Discussion

In this paper, we present new empirical evidence on how data privacy concerns shape people’s usage of climate-relevant applications. Through three studies with a nationally representative UK sample, we find behavioural patterns consistent with the privacy paradox and extend its explanatory value by uncovering more complex causal pathways in individuals’ risk calculus. Our findings show that although data privacy concerns are widespread and increasing, they do not uniformly reduce the use of climate-relevant applications. Crucially, privacy concerns do not act as direct deterrents, but rather through perceptions that applications collect and utilise personal data.

Our experimental results from Study 1 show that when AI involvement is made explicit, willingness to use climate-relevant applications declines, particularly when their perceived relative advantage is low. This effect is most pronounced in areas such as online retail and smart energy systems. Two explanations for continued usage despite privacy concerns is that many AI-enabled applications are not yet perceived as posing significant privacy risks, or that the AI component of their functionality is not salient to users. Our results align with the privacy calculus model, according to which individuals weigh perceived benefits against perceived privacy risks^41,45,46^.

Building on these findings, Studies 2 and 3 further show that individuals with strong privacy concerns are more engaged with climate-relevant applications if they implement data protective behaviours. This mediation effect suggests that rather than avoiding applications, users actively manage their privacy concerns through data-management practices. In addition, Study 2 found that familiarity with AI, reflected in proactive engagement such as seeking information or using GenAI tools, was significantly associated with lower perceived AI risks. This likely reflects a familiarity or desensitisation effect, whereby greater exposure to AI normalises its use, reduces ‘fear of the unknown’ and reduces perceived risk. These findings offer a behaviourally grounded explanation of the privacy paradox to explain why concerns do not always translate into disengagement. Our findings add new empirical weight to this theory and provide evidence for its relevance in the climate-AI nexus.

Our findings also introduce a behavioural dimension that complements existing AI risk frameworks, such as the MIT AI Risk Repository^47^, which primarily catalogue technical, operational and societal risks. Although such frameworks focus on system-level mitigation strategies, our results suggest that users themselves also play a role by actively managing privacy concerns through data protective behaviours that are associated with greater uptake of climate-relevant applications. Although the protective behaviours studied are not unique to AI, they are integral to the same data ecosystems on which AI-enabled applications depend and therefore provide insight into how users manage privacy risks in increasingly data-intensive services.

However, the limits of behavioural coping are also evident. Residual negative effects of privacy concerns on the use of bike-share schemes and meal kits suggest that even proactive users cannot fully compensate for opaque data practices or mandatory data sharing embedded in platform design. Public scrutiny of bike-sharing services such as Ofo and Mobike for extensive geolocation tracking without clear user consent^48,49^, and regulatory action against HelloFresh for misuse of customer data^50,51^, illustrate how structural features of data governance shape user responses. These cases reinforce that privacy concerns are grounded in experience and highlight the importance of enforceable transparency and meaningful user agency. Clear consent and openness about data use are essential^34,46^, but must be accompanied by real choice, such as the ability to limit, refuse or tailor data sharing, to ensure that transparency translates into control.

These findings have implications for ongoing debates about AI governance. Numerous AI governance frameworks already exist internationally and increasingly advocate for AI to support sustainability goals^32,33^. Our findings suggest that maintaining attention to data privacy and meaningful user agency is essential to achieving these goals. Many regulatory approaches developed for data-driven digital services, such as transparency requirements and user-centred consent design, remain applicable in AI-enabled contexts. Existing policy frameworks increasingly extend general data governance principles to AI systems. For example, the OECD’s AI policy guidance emphasises that longstanding data protection principles apply directly to AI systems^52^, and the EU Artificial Intelligence Act (Regulation (EU) 2024/1689) similarly extends established transparency and accountability requirements to AI contexts. While AI presents additional regulatory challenges, our findings indicate that strengthening and enforcing existing governance tools, particularly those addressing data collection and user control, may be more effective than constructing entirely new behavioural frameworks specific to AI. This includes regulatory measures that extend beyond transparency alone, such as enforceable requirements on data minimisation, purpose limitation, and data protection by design^32,33,53–55^.

Beyond immediate governance implications, our findings also point to two priorities for future research. First, following Bossert and Loh^56^, we argue for holistic sustainability assessments of AI, including its social acceptability, data practices and demands, and perceived intrusiveness. Addressing such dimensions would help identify trade-offs and conflicting interests. Second, future research should directly measure perceived data collection across applications to better triangulate practices with perceptions. These two lines of enquiry would clarify not just whether AI should be used in each context but whether it can be used in ways that people trust and are willing to sustain.

To address data-related privacy concerns in practice, service providers should prioritise transparency, accountability and meaningful user agency over data collection and use. Complementing these provider-side obligations, our findings identify three behavioural strategies that could help manage data privacy concerns and support engagement with climate-relevant applications:

Utilise the privacy paradox by emphasising the relative advantage of applications, highlighting both climate (pro-social) and personal (functional) benefits to outweigh privacy concerns.

(2) Build digital privacy literacy: Equip users with the knowledge and tools to protect their data, enabling confident and informed engagement without opting out entirely.

(3) Promote informed familiarity: Increase transparent public communication and awareness of AI systems.

These strategies are increasingly pertinent given recent evidence on the widespread undisclosed data tracking by 100 leading Android apps^57^. Without responsible data governance, AI-enabled services risk eroding public trust^58^—even when they serve climate goals. To scale climate-relevant applications equitably and sustainably, our findings support calls for developers to incorporate ‘privacy by design’ principles, ensuring responsible data collection and usage. As results from Study 1 demonstrated, perceived relative advantage of an application is important in user decision making. Effectively communicating tangible user benefits and offering strong privacy as a unique selling proposition (USP) could help unlock wider public uptake of climate-relevant applications. Within a diverse ecosystem of AI application providers, those with a strong privacy (USP) can differentiate themselves and build user trust. As discussed in recent literature on LLMs^34^, our results also support the need for: AI developers to implement rigorous frameworks to protect user privacy and ensure fairness; policymakers to mandate transparency and user control as defaults; and global governance effort such as those led by the Global Partnership on AI, to ensure privacy, equity and environmental alignment^59^.

Broader implications emerge from our research. While attention to existential or otherwise extreme risks of AI is growing^60–63^, our findings advocate for incorporating a more scientifically grounded and broader range of social considerations into AI application development. Such considerations are critical for aligning AI systems with climate mitigation goals and promoting low-carbon lifestyles.

Overall, our research demonstrates that AI data collection practices and individuals’ data privacy concerns play an influential role in shaping engagement with AI-enabled, data-driven applications with potential climate benefits. Although privacy concerns are found to be widespread, they do not uniformly deter use. Instead, perceptions of data collection influence willingness to engage, mediated by data protective behaviours and perceived user benefits. Familiarity with AI, reflected in proactive engagement such as seeking information or using generative AI tools, was found to be associated with lower perceived risks. These findings contribute to the privacy paradox by showing how users actively manage privacy trade-offs when interacting with data-driven services.

The results support the need to build public trust in AI-enabled climate solutions through transparent data use, genuine user control, and accountability by developers. Embedding such principles alongside clear communication of both personal and societal benefits could help ensure that AI supports climate goals in ways that people understand, value, and are willing to adopt.

## Methods

### National online survey

We conducted three studies to investigate how data privacy concerns influence the use of climate-relevant applications, as well as the factors shaping this relationship. Data were collected through a nationally representative survey of UK adults (*n* = 2078) for which participants received monetary compensation. Sample demographics are available in Supplementary Table 14. The survey instrument was administered by a market research company (Qualtrics) in April 2024 with a median duration of 11.9 min. The UK was chosen as the reference country due to the wide availability of climate-relevant applications and the policy imperative to reduce per capita emissions in line with legally binding domestic climate targets^64^. The research was approved by the University of Oxford’s School of Geography and the Environment, Departmental Research Ethics Committee, reference number SOGE1A2021-233. All participants gave informed consent before participating in the online survey used in the studies. No personally identifying data were collected or retained.

The survey instrument comprised six blocks of questions followed by a vignette-based experimental survey block (Table 2). Questions used either single or multi-item scales based on precedents in the literature (with slight modifications to fit the current research context) and newly developed items. Many questions consisted of statements with level of agreement captured by a 5-point Likert scale. The survey instrument provided in Data Availability outlines which questions are from precedent literature.

**Table 2:** Survey blocks and example questions

Block	Topic	Example questions
1	Socio demographics and household characteristics	What is the highest level of education you have completed?
2	Application usage status and change in usage (x15 applications)	How often do you do the following [domain]-related activities? Compared to a year ago, how would you describe the frequency you do the following activities?
3	Application usage intention (1 application x 4 domains)	Within the next year, how likely are you to use [application]?
4	Technology attitudes	To what extent do you agree or disagree with the following statement…? Often it is easier to do things without using digital technologies.
5	Data privacy concerns and change in concern	When using the internet in daily life, how concerned are you that… Your internet usage information (including details of items you searched or purchased) is shared with websites or companies which you don’t use? Has your level of concern changed compared to a year ago for the following…?
6	AI exposure and perceptions	How important have these sources of information been in shaping your opinion of AI? To what extent do you agree or disagree that AI will benefit you?
Vignette – participants randomly allocated to one block (application domain – salience of AI collecting personal data)		
7	Retail—High	Based on the information provided in the story, how likely are you to use a [application] like the one described?
8	Retail—Low	To what extent do you agree or disagree with the following statements about [application] like the one described? Using them would be better than other available options I think they would be easy to use I think they would be useful When using them, they would collect personal data
9	Mobility—High	
10	Mobility—Low	
11	Food—High	
12	Food—Low	
13	Home—High	
14	Home—Low	

Initial survey blocks captured demographic and contextual characteristics to inform inclusion criteria (above the age of 18) and quota fulfilment (nationally representative sample on age and gender), followed by self-reported use of a diverse set of 15 data-driven climate-relevant applications largely enabled by AI (e.g. learning algorithms, cloud-based services, or other forms of data-dependent service-provision) in four domains of daily life: retail, mobility, food and home energy (Fig. 3).

These applications are referred to as ‘climate-relevant’ throughout to denote applications whose design and use have implications for energy demand and emissions, recognising that their ultimate climate impact depends on user behaviour, system interactions, and potential rebound effects. Logic branching allowed follow-ups regarding usage frequency to be customised according to current, past or non-use. For example, a participant who indicated current use of an application was then asked, ‘how often do you do [activity]?’. Subsequent blocks assessed usage propensity for four case study applications (one per domain), general attitudes towards technology^65^, data privacy concerns^66,67^, data protection behaviours^68,69^ and perceptions of and attitudes to AI^25^. The latter was taken directly from the Office for National Statistics (ONS) survey on AI and included a measure of perceived AI risk which did not specify a particular type of risk. This block was positioned after questions on data privacy concerns and protection behaviours to provide contextual grounding in data-related issues. Several items on AI information sources were adapted from ref. ^35^, and additional questions included, for example to assess AI familiarity e.g. usage of GenAI.

To examine the potential effects of the recent surge in AI awareness following the release of ChatGPT 3.5, we also included retrospective items measuring changes in attitudes and behaviours over the previous year of 2023 (the first full year of the GenAI hype). To capture change in application usage, we asked ‘Compared to a year ago, how would you describe the frequency you do the following activities?’.

Following the main survey, we implemented a vignette experiment to test how awareness of AI data practices affects perceptions of climate-relevant apps that use AI and their usage propensity. The vignettes represented stylised but plausible versions of existing or emerging applications rather than descriptions of specific commercial products. They were designed to capture perceptions of AI-related data practices across realistic, recognisable domains (e.g. smart thermostats, bike-sharing, food-waste apps) while maintaining experimental control. To ensure realism, we mapped each vignette to a representative market leader and reviewed their publicly available documentation on ownership, business model, data collection, and AI use. As summarised in Supplementary Table 1, these leading services (e.g. Lime, Too Good To Go, Nest, eBay) collect similar classes of user data such as identifiers, location, device telemetry, and transactional records, primarily for service delivery, analytics, and optimisation, often explicitly referencing machine-learning or AI techniques. This mapping grounds the plausibility of our scenarios and contextualises the privacy attributes experimentally varied in this study.

Each respondent was randomly assigned to one of the eight vignettes shown in Table 3, describing the use of one of four climate-relevant applications, with either an increased or reduced emphasis on personal data being collected by AI. The vignettes respected the principles of realism, clarity, simplicity and internal consistency^70,71^.

**Table 3:** Vignettes used in Study 1

Climate-relevant applications	High salience of AI collecting personal data	Low salience of AI collecting personal data
P2P retail platform	As Alex scrolled through the listings on the online marketplace, ready to buy a used camera, a new feature caught their eye. The platform now integrated advanced AI algorithms to help buyers optimise their searches. Intrigued, they decided to give it a try. Little did they realise that the AI was not only assisting with search details but also collecting and analysing data about their past transactions and preferences. A notification popped up, briefly explaining how this personalised AI assistance would enhance their purchasing experience. Reflecting on the efficiency of the system, Alex pondered whether the benefits of improved searches were worth the potential implications of sharing such personal purchasing data.	As Alex decided to buy a used camera, they navigated the familiar process of scrolling through the listings on the online marketplace. The platform had recently introduced a user-friendly interface, making it easier to search for listings. A subtle message briefly appeared, mentioning some backend improvements for a smoother buying experience. Engrossed in the simplicity of the process, Alex wondered about the balance between technological enhancements and the straightforward joy of buying unwanted items.
Bike share scheme	As Chris unlocked their bike from the city’s bike share scheme, they noticed a new feature on the app prompting them to enable AI-powered route recommendations for a more efficient journey. Intrigued by the prospect of faster routes, they consented without realising that the AI was also gathering data on their cycling habits and travel patterns. A notification popped up, briefly explaining how AI assistance would enhance their biking experience. Reflecting on the convenience of optimised routes, Chris pondered whether the benefits of efficient cycling were worth the potential implications of sharing such personal data.	As Chris unlocked a bike from the local bike share scheme, they appreciated the simplicity of the process, navigating the app’s easy to use interface to find a nearby docking station. Recently, the app had undergone some updates, promising a smoother experience for users. A subtle message briefly appeared, mentioning backend improvements for a more seamless biking experience. Engrossed in the ease of renting a bike, Chris wondered about the balance between technological enhancements and the straightforward joy of cycling around the city.
Food waste reduction app	As Charlie explored a food waste reduction app to rescue surplus food from local businesses, they noticed a new feature using AI to suggest nearby establishments with excess food available for purchase. Intrigued by the opportunity to reduce food waste, they enabled the feature without realising that the AI was also collecting data on their food preferences and consumption habits. A notification popped up, briefly explaining how AI assistance would enhance their experience in rescuing surplus food. Reflecting on the efficiency of connecting surplus food with those in need, Charlie pondered whether the benefits of reducing food waste were worth the potential implications of sharing such personal data.	As Charlie browsed a food waste reduction app to find surplus food from nearby businesses, they appreciated the straightforward process of rescuing edible items that would otherwise go to waste. The app had recently undergone some updates, streamlining the user experience for both rescuers and participating establishments. A subtle message briefly appeared, mentioning backend improvements for a more efficient food rescue experience. Engrossed in the simplicity of finding and rescuing surplus food, Charlie wondered about the balance between technological enhancements and the straightforward joy of reducing food waste.
Smart thermostat	As Jordan engaged with their new smart thermostat, they marvelled at its ability to intuitively adjust the temperature based on their preferences. Unknown to them, the device was meticulously gathering data on their daily routines, learning when they preferred cosy or cool atmosphere. A notification popped up, shedding light on the thermostat’s data utilisation for personalised climate control. Reflecting on the complexities of AI and data, Jordan pondered whether the convenience of a perfectly tailored environment was worth the potential implications of sharing such personal information.	As Jordan explored the features of their new smart thermostat, they were fascinated by its seamless adjustments to maintain a comfortable temperature. Little did they know, the device was quietly adapting to their daily routines, effortlessly creating the ideal atmosphere they desired. A notification appeared briefly, mentioning the thermostat’s capabilities for personalised climate control. Contemplating the convenience of a perfectly tailored environment, Jordan wondered about the balance between technological advancements and personal comfort.

A pilot survey (*n* = 56) was used with two additional exploratory questions to identify applications with varying perceptions of data collection and associated concerns, informing vignette application selection (Supplementary Table 15). Quota sampling was used to approximate 250 respondents per vignette to ensure sufficient statistical power. Final sub-sample sizes ranged from 245 to 275. To elicit accurate estimates of usage propensity, respondents were asked post-vignette whether they would use the application described, rather than assessing what others should do^70^.

Additional post-vignette questions were informed by prior research to investigate mechanisms of the privacy paradox by assessing respondents’ perceptions of personal data collection during application use, alongside perceived relative advantage (operationalised as a latent construct using three items: better than alternatives, easy to use, and useful; see Fig. 3 and Supplementary Fig. 1). Respondent sub-sample characteristics are available in Supplementary Table 16.

### Data preparation

A total of 8920 respondents started the survey and consented and committed to taking part. 5478 respondents were disqualified with the first attention check, and 794 respondents were disqualified at the second attention check which assessed attentiveness while reading the vignette. From the soft launch (*n* = 100), the median time for completion was 9.41 min. A speed checker of half this median (4.69 min or 281 s) was implemented for the full launch to automatically terminate those who did not responding thoughtfully. This speed checker removed 406 respondents. Overall, 525 completes were removed by Qualtrics due to straight-lining, speeders (406), likely bots (5) or duplicate responses (79). Qualtrics delivered a file with 2123 completes from which verification and quality checks were conducted by researchers. 45 responses did not include question blocks 9–16 and were therefore removed. The remaining were determined as high quality, resulting in a final sample of *n* = 2078.

We conducted data reduction and preparation steps for all three studies. Reliability testing demonstrated acceptable internal consistency for key scales: *perceived relative advantage* (*α* = 0.784), *data privacy concerns* (*α* = 0.878), and technology attitudes—referred to as *technophilia* (*α* = 0.705). *Data protection behaviour* items showed marginal internal consistency (*α* = 0.699). *Perceived relative advantage* items were combined into a latent variable for SEM, while *data privacy concerns* and *technophilia* scores were summed following established approaches^66–68^. Notably, scale items for *technophilia* were recoded where necessary to ensure consistent directional meaning^68^. *Data protection behaviours* were summed to create a scale.

For each AI application, respondents who stated, ‘used in the past but not now’ or ‘never used’, were recategorised as ‘never’ for *usage frequency*. Ordinal variables representing change over the past year were constructed for *data privacy concerns* and *application usage*. For *data privacy concerns*, change scores were created by summing responses across six items (*privacy_compare 7.2_1* to *7.2_6*), yielding a scale *‘change data privacy concerns’* from – 6 (large decrease) to +6 (large increase). The variable ‘*change application usage’* was derived by combining responses from current users’ reported changes in frequency over the past year (*compare_[application]*), and past users’ discontinuation timelines (*lastused_[application]*), i.e. capturing past users who discontinued during the past year.

To maintain analytical clarity, ‘don’t know’ responses were recategorised based on variable type

- For current *application usage*, and *data protection behaviours*, ‘don’t know’ responses were recoded as ‘never done’, based on the assumption participants unable to recall engaging in a behaviour or with an application likely had not done so recently or meaningfully.
- For *technophilia*, ‘don’t know’ responses were recategorised as ‘neither agree nor disagree’ preserving the ordinal scale structure and avoid unnecessary missingness.
- For *perceived AI risks*, *usage propensity* and *usage frequency*, ‘don’t know’ responses were labelled as missing data and regression imputation was used to deal with missing data and to facilitate bootstrapping in the SEMs.

### Data analysis

We ran statistical analyses separately for each study using SPSS (version 30.0.0.0) and Amos Graphics (version 29.0). For Study 1, to verify participants were randomly assigned across the eight vignette groups and assess baseline differences across socio-demographic variables and technology attitudes, Kruskal-Wallis tests were used for continuous variables e.g. *technophilia*, and chi-square tests for nominal variables e.g. *education level*. No significant differences were detected (Supplementary Table 16).

Wilcoxon signed-rank tests assessed changes in application *usage propensity* (measured pre- and post-vignette exposure). Mann-Whitney U tests compared differences in *usage propensity* changes, as well as differences in *perceived data collection* between high and low AI salience conditions.

Causal pathways between *data privacy concerns* and application *usage propensity* were examined through SEMs which incorporated *perceived relative advantage* and *perceived data collection*. The overarching model was specified using the privacy paradox as a heuristic framework to explore discrepancies between individuals’ stated privacy concerns and their actual technology use and was applied to the four case study climate-relevant applications. Both measurement and structural components were included, with a latent construct derived from three observed indicators of *perceived relative advantage*. The scale was fixed by constraining the variance to 1. SEMs were estimated using the covariance matrix, applying the maximum likelihood (ML) procedure with bootstrapping to account for non-normality (Shapiro-Wilk *p* > 0.05).

Model fit indices show acceptable to excellent fit across application models (Supplementary Table 4). The specific application models for food waste reduction app (RMSEA = 0.059, CFI = 0.978) and smart thermostat (RMSEA = 0.069, CFI = 0.974) demonstrated excellent fit. Fit was weaker for the P2P retail platform and bike-share scheme (RMSEA > 0.09), though still within acceptable thresholds suggesting models fit the data very well^72^.

For Study 2, descriptive statistics were produced for independent variables (Supplementary Table 6), and for participant subgroups based on their *perceived AI risk* (Supplementary Table 5). Spearman’s rank correlations were used to explore associations between variables (Supplementary Table 7); monotonicity was confirmed through preliminary visual checks. Mann-Whitney U tests assessed group differences in *data privacy concerns* between current users and non-users of each of the four case study applications.

SEM was conducted per application to investigate the mediation of *data protection behaviours* between *data privacy concerns* and application *usage frequency*. ML with bootstrapping based solely on observed variables was used. However, SEM model fit indices indicated relatively poor fit: RMSEA values ranged from) to 0.144 (bike-share scheme), CFI values from 0.862 to 0.614, and TLI values from 0.724 to 0.228 (Supplementary Table 10).

The models’ RMSEA values indicate a reasonable fit for two of the applications (P2P retail platform, 0.036 and food waste reduction app, 0.079), but the low CFI and TLI scores across the four models (Supplementary Table 10) suggest that the specifications did not fully capture the underlying data structures. The results should therefore be interpreted as indicative.

For Study 3, we applied the Spearman’s rank correlations and SEM analyses from Study 2 to an additional 11 climate-relevant applications, providing insights across all 15 studied applications. Results addressing our research questions (Fig. 1) are reported in the main text, with the Supplementary Information providing correlation coefficients (Supplementary Table 17), model results (Supplementary Table 12) and robustness and sensitivity checks (Supplementary Table 13).

## Supplementary information

Supplementary Information.

## Data Availability

The dataset generated by the survey research during the current study is available to download at: https://reshare.ukdataservice.ac.uk/858386/.
