# Supplementary material for: Data concerns undermine AI applications for climate
Source: NPJ Clim Action. 2026 Jun 6;5(1):81. doi: 10.1038/s44168-026-00390-2 (PMC13572199; doi:10.1038/s44168-026-00390-2)
Supplement: Supplementary file 1 [file 44168_2026_390_MOESM1_ESM.pdf]

Supplementary Information

Supplementary Table 1. Examples of market leader characteristics, data practices and use of AI/ML for the 15 applications investigated. Market leaders for the four case study applications from Study 1 and 2 are highlighted in bold with links to resources underlined.

Domain	Application (UK market leader)	Ownership & business model	Main data collected (examples)	How data are used (vendor-stated)	Sharing / retention (T&Cs / privacy page)	Explicit ML / AI mention (publicly available)
Retail	Online large retailers -Amazon	Public multinational. Revenue: product sales, marketplace fees, advertising, and subscription services.	Personal identifiers, payment and billing details, order and item history, browsing and click logs, device/IP data, delivery addresses.	Fulfil orders, personalise recommendations, target advertising, detect fraud, optimise logistics, conduct analytics.	Cross-affiliate processing, sharing with third-party processors, retention for business/legal reasons and customer rights. (Amazon)	Developing and deploying state of the art AI and machine learning models to power customer experiences for both customer-facing services and internal operations e.g., personalised shopping recommendation engines, AI-powered robots that optimize order fulfilment in warehouses. (Amazon AI)
	**P2P retail platform -eBay**	Public company. Revenue: listing and transaction fees, advertising, and payment processing.	Personal identifiers, payment and billing details, transaction and listing data, messages, device/IP data.	Fulfil orders, Personalisation, search ranking, fraud detection, platform integrity, analytics.	Sharing with eBay affiliates and third-party providers and services, retention for business/legal reasons; customer rights and safety. (eBay)	Use AI-powered tools and products to improve services, offer new enhanced features (e.g. list generation, image background enhancement, AI-assistance, analyse data, personalisation, support fraud detection. Explicitly references using personal data to train/test/validate AI models and third party models (eBay and AI)
	Online small retailers -Etsy	Public company. Revenue: seller fees, transaction commissions, and advertising.	Personal identifiers, payment and billing details, account and order history, buyer/seller messages, device/IP data, listing metadata.	Maintain accounts, enable transactions, payments, recommendations, analytics, and marketplace operations.	Sharing with service providers, law enforcement authorities and following GDPR rights (deletion, access). (Etsy)	Use AI tools behind the scenes for search, recommendations, and image-based matching, offers a "Writing Assistant" AI tool and uses machine learning to detect policy violations. (Etsy News)
Mobility	Car clubs -Zipcar	Wholly owned subsidiary of a public company. Revenue: memberships, usage fees, and subscription plans.	Personal identifiers, driving licence details, booking and usage logs, GPS/telemetry, payment tokens.	Manage bookings, billing, vehicle access and security, insurance, compliance, analytics.	Sharing with affiliates/third-party providers, rights to request access/erasure. (Zipcar)	Use third party ML-powered fleet optimisation tool (Autofleet) to maximise availability and fleet efficiency. (Zipcar Autofleet)
	P2P car sharing -Turo	Private company. Revenue: service fees from vehicle owners and renters.	Personal identifiers, driving licence and background check data, trip logs, GPS/usage data, payment information.	Verification, bookings, insurance and claims handling, fraud detection, analytics.	Background checks, sharing with processors and insurers; retention per legal/operational needs. (Turo)	Use third party ML and augmented intelligence (DataRobot) for pricing, risks levels and marketing decisions (Turo DataRobot). Use a global advanced AI agent (Turo Voiceflow).
	Ride sharing -Uber	Public company. Revenue: fares, commissions, delivery fees, and advertising.	Personal identifiers, trip geolocation, payment data, driver and vehicle documents, device and sensor telemetry.	Ride and delivery operations, mapping, dispatch, safety, fraud detection, analytics.	Cross-country transfers, sharing with service providers/regulators; rights and data request procedures in place. (Uber)	Use an internal ML platform 'Michelangelo' for its central operations including ETA prediction, river-driver matching, fraud detection and content ranking. (Uber Michelangelo)
	**Bike share scheme -Lime**	Private company. Revenue: per-ride fees and municipal contracts.	Personal identifiers, precise GPS location during rides, ride telemetry, device/browser data, payment tokens.	Unlock/locate vehicles, billing, fleet rebalancing, safety analytics, operational optimisation.	Third-party processors, sharing with city partners, and retention practices; opt-out of location limits service. (Lime)	Use an internal AI/ML computer vision platform 'Lime Vision' for safety features e.g., to detect sidewalk riding in real time. (Lime Vision)
Food	Online grocery shopping -Ocado (UK)	Public company. Revenue: online grocery sales, logistics, and retail technology licensing.	Account details, order and basket history, delivery address, browsing activity, device/IP data, payment tokens.	Order fulfilment, delivery logistics, personalisation, marketing, analytics.	Sharing with processors/partners, retention, rights to object/erase. (Ocado)	Embeds AI in over 100 applications including webshop personalisation, robotic fulfilment, predictive demand forecasting and route optimisation (Ocado Smart Platform)

Domain	Application (UK market leader)	Ownership & business model	Main data collected (examples)	How data are used (vendor-stated)	Sharing / retention (T&Cs / privacy page)	Explicit ML / AI mention (publicly available)
	Online meal delivery -Deliveroo	Public company. Revenue: delivery fees, restaurant commissions, and advertising.	Account and order history, delivery address, device/IP data, payment information.	Match customers to restaurants, dispatch riders, handle payments, prevent fraud, perform analytics.	Sharing with third party partners and processors; cookie/app consent controls. (Deliveroo)	Use an internal ML platform using open-source tools (Kubernetes, Argo and Metaflow) for key service features e.g., personalised recommendation, fraud detection, order dispatch, order prediction. (Deliveroo ML platform)
	Meal kits - HelloFresh	Public company. Revenue: subscription meal-kit sales.	Account data, delivery address, purchase/subscription history, payment data, dietary preferences.	Behaviour analytics, fulfil subscriptions and deliveries, personalise offers, customer service, advertising.	Sharing with third-party partners and processors, law enforcement, data retained for six years. (HelloFresh)	Use over 1500 models weekly across marketing, recommendation and operational systems (HelloFresh Group). Use Tecton to standardise ML across the organisation (Tecton), and Sift to reduce promo abuse (Sift)
	**Food waste reduction app - Too Good To Go**	Private B-Corp. Revenue: commission on partner transactions.	Account information, geolocation, order history, partner/store data.	Match users with nearby surplus food, process payments, send notifications, perform analytics.	Sharing with third-party partners/payment processors and GDPR rights (deletion/access). (TooGoodToGo)	AI powered discounting, expiry date prediction, and retailer inventory optimisation (TooGoodToGo News). Employs Mistral AI models to analyse retailer partner retention (Mistral AI AWS).
Home	**Smart thermostat -Google Nest Learning Thermostat**	Subsidiary of a public company. Revenue: device sales and digital ecosystem integration.	Device telemetry (temperature, schedule), occupancy/presence data, energy usage, account information.	Automate heating schedules, provide energy reports, enable remote control, improve device performance.	Device/cloud processing, Google Account integration, and options to manage/delete data via account settings. (Nest)	Use self-learning algorithms to infer homeowner schedules and optimise heating/cooling. Implicitly ML/algorithmic inference (Nest)
	Learn about energy use -Octopus Energy	Private company. Revenue: energy supply, smart tariffs, and digital services.	Meter and energy-usage data, account information, smart device metadata, app interaction data.	Provide consumption dashboards, billing, personalised tips, tariff recommendations.	Energy-use data and retention for billing/regulatory reasons, sharing with service partners. (Octopus Energy)	No clear public evidence was found that Octopus offers live, ML-driven user feedback on current energy usage.
	Learn how to reduce energy use - Octopus Energy	Private company. Revenue: energy supply, smart tariffs, and demand-management services.	Smart meter readings, device data, behavioural survey inputs.	Provide personalised energy-saving advice and usage insights.	Usage of meter data to provide services and recommend measures. (Octopus Energy)	Use an internal ML 'Kraken' for tariff decisioning and optimisation. (Octopus Kraken)
	Shift time of energy use -Octopus Energy	Private company. Revenue: dynamic smart tariffs and flexibility-market participation.	Near real-time meter data, device schedules (EVs, heat pumps), tariff selection data.	Automate smart-device scheduling, optimise pricing, manage billing and settlement.	Usage of meter data for billing/market settlement and use in smart-tariff operation, retention for regulatory purposes. (Octopus Energy)	Use an internal ML 'Kraken' to automate optimal charging times and demand flexibility e.g., virtual power plant. (Octopus Virtual)

Supplementary Table 2. Wilcoxon signed-rank tests were conducted to determine the effect of the vignette on usage propensity, which was measured pre and post vignette.
The difference scores were approximately symmetrically distributed, as assessed by a histogram with superposed normal curve. There was a statistically significant median decrease in usage propensity for participants exposed to high AI salience vignettes from pre-vignette to post vignette z = -5.197, p <0.001. Mann-Whitney U tests were then conducted to determine if there were differences in the propensity change between participants exposed to high and low AI salience.

			Pre-vignette		Post-vignette		Within group analysis					Between group analysis - Propensity change					
**Application**	**Vignette exposure**	**n**	**mean (SD)**	**median**	**mean (SD)**	**median**	**mean diff.**	**median diff.**	**z**	**P (2-tailed)**	**effect size r**	**mean rank score**	**median propensity change**	**U**	**z**	**P (2-tailed)**	**effect size r**
**All**	**High**	1035	2.86 (1.494)	3	2.60 (1.330)	3	-0.260 (1.476)	0	-5.197	**<0.001**	**-0.161**	966.55	0	464244.50	-3.625	**<0.001**	**- 0.081**
	**Low**	985	2.85 (1.481)	3	2.85 (1.322)	3	0.004 (1.231)	0	0.294	0.769		1056.69	0				
**P2P retail platform**	**High**	241	3.47 (1.411)	4	2.66 (1.232)	3	-0.809 (1.731)	-1	-6.397	**<0.001**	**-0.412**	218.66	-1	23436.50	-3.260	**0.001**	**-0.149**
	**Low**	235	3.50 (1.357)	4	3.22 (1.109)	3	-0.285 (1.300)	-1	-3.169	**0.002**	**-0.207**	258.84	0				
**Bike share scheme**	**High**	273	2.13 (1.360)	2	2.19 (1.341)	2	0.055 (1.240)	0	0.749	0.454		245.98	0	29751.50	-1.965	**0.049**	**-0.087**
	**Low**	240	1.91 (1.227)	1	2.13 (1.291)	2	0.217 (1.044)	1	3.256	**0.001**	**0.210**	269.54	0				
**Food waste reduction app**	**High**	259	2.80 (1.421)	3	2.76 (1.320)	3	-0.046 (1.386)	0	-0.501	0.616		248.62	0	30723.50	-1.274	0.203	
	**Low**	253	2.79 (1.388)	3	2.90 (1.286)	3	0.107 (1.209)	0	1.706	0.088		264.56	0				
**Smart thermostat**	**High**	262	3.13 (1.453)	3	2.83 (1.326)	3	-0.294 (1.396)	0	-3.084	**0.002**	**-0.191**	250.54	0	31188.00	-1.512	0.131	
	**Low**	257	3.18 (1.454)	3	3.14 (1.305)	3	-0.031 (1.274)	0	-0.481	0.631		269.65	0				

Supplementary Table 3. Differences in perceived data collection between vignettes with high AI salience and low AI salience. Mann-Whitney U test results with effect sizes.

	High AI salience vignette		Low AI salience vignette					
**Application**	**n**	**mean rank score**	**n**	**mean rank score**	**U**	**z**	**P (2-tailed)**	**effect size r**
**All**	1057	1102.48	1021	974.30	606167.50	5.169	**<0.001**	**0.113**
**P2P retail platform**	250	265.83	246	230.89	35082.50	2.946	**0.003**	**0.132**
**Bike share scheme**	275	286.84	245	230.93	40931.00	4.475	**<0.001**	**0.196**
**Anti food waste app**	264	274.88	262	252.99	37598.50	1.744	0.081	
**Smart thermostat**	267	277.54	268	258.49	38326.50	1.504	0.133	

Supplementary Table 4. Study 1's goodness of fit indices for the four structural equation models

					Model fit indices			
**Application**	**n**	**$\chi 2$**	**p**	**d.f.**	**RMSEA**	**CFI**	**TFI**	**AIC**
**Recommended value**					<0.080	>0.90	>0.90	
**P2P retail platform**	476	47.827	<0.001	8	0.102	0.938	0.883	85.827
**Bike share scheme**	513	45.175	<0.001	8	0.095	0.940	0.887	83.175
**Anti food waste**	512	22.171	0.005	8	0.059	0.978	0.959	60.171
**Smart thermostat**	519	27.565	0.001	8	0.069	0.974	0.951	65.565

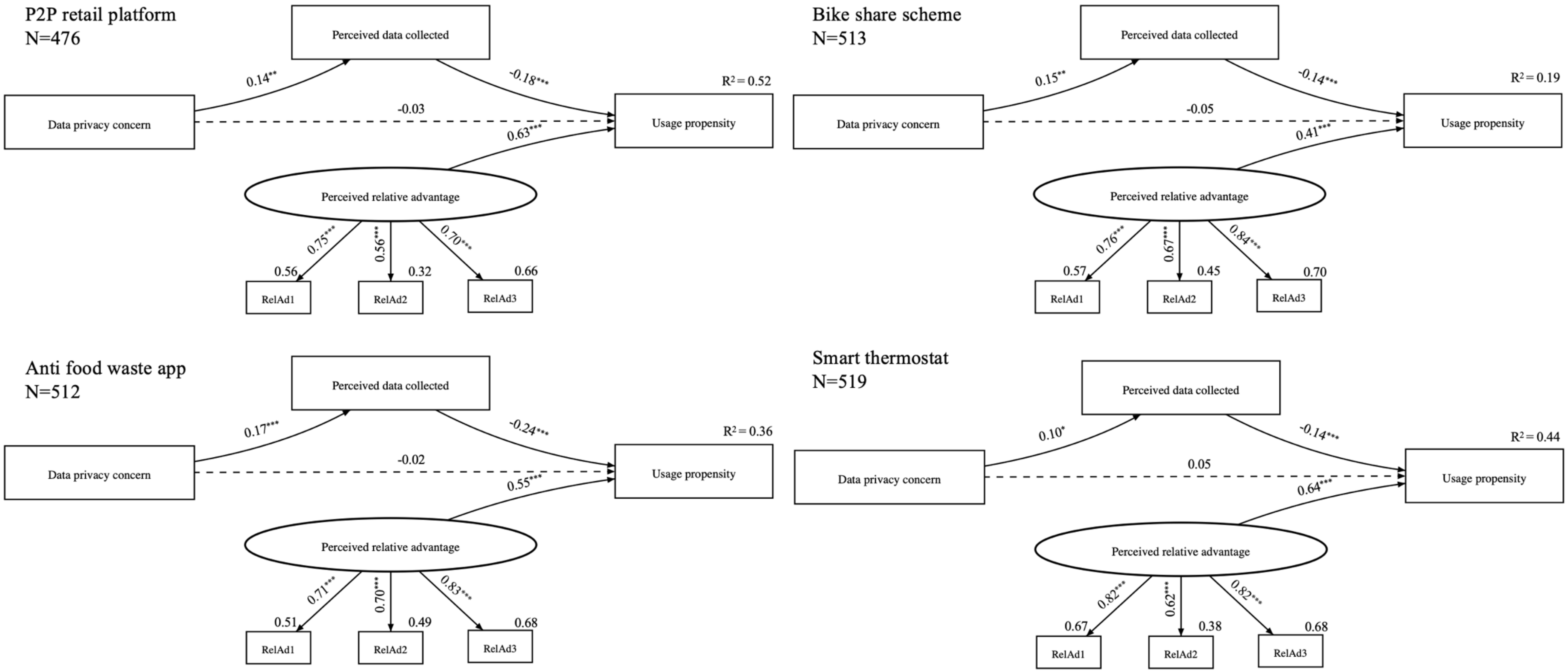

Supplementary Fig. 1. Structural equation model showing the direct and indirect effects of data privacy concerns, perceived data collected and relative advantage on usage propensity. The latent variable 'perceived relative advantage' consists of: RelAd1 'better than alternatives', RelAd2 'easy to use' and RelAd3 'useful'. Numbers adjacent to the arrows are standardised path coefficients and indicative of the effect size of the relationship. Dashed lines indicate non-significant relationships. *P<0.05, **P<0.01 and ***P=<0.001. R^2 represents the proportion of variance explained by the relations in the Maximum Likelihood path model.

Supplementary Table 5. Personal characteristics of survey respondents grouped by their perceptions of AI risks and benefits.

Variable		More benefits than risks n=351	Equal benefits and risks n=910	More risks than benefits n=720
**Gender**	Female	41.0	52.0	54.4
	Male	59.0	47.9	45.0
**Age**	18-34	38.2	32.1	21.6
	35-54	35.9	29.1	28.9
	55+	25.9	38.9	49.5
**Education**	Secondary or lower	8.0	8.2	8.3
	College or other	35.0	47.9	51.5
	Undergraduate or higher	56.4	42.5	39.9
		**Mean (SD)**	**Mean (SD)**	**Mean (SD)**
**Technology attitudes and use**	Technophilia score	21.16 (4.50)	18.88 (3.67)	16.58 (4.06)
	Number of applications currently used	7.49 (3.54)	6.38 (2.85)	5.54 (2.52)
	Current data privacy concerns (sum)	17.75 (6.76)	18.22 (6.04)	20.36 (6.19)
	Change in data privacy concern (sum)	1.84 (2.04)	1.92 (2.01)	2.63 (2.18)
**Perceived AI impacts**	Personal positive impacts (sum)	3.37 (2.08)	2.36 (1.86)	1.1 (1.52)
	Personal negative impacts (sum)	2.73 (1.88)	3.38 (1.86)	4.32 (2.07)
**Perceived AI wider impacts on**	…the economy	3.35 (1.05)	3.22 (1.02)	2.89 (1.35)
	…the society	3.21 (0.90)	2.82 (0.97)	2.07 (0.99)
	... your quality of life	3.57 (0.97)	3.76 (1.17)	3.29 (1.57)
	... medical research and more	3.53 (0.84)	3.39 (0.80)	3.03 (1.01)
	... the efficiency of energy use	3.5 (0.89)	3.34 (0.91)	3.18 (1.18)
	... climate change	3.58 (1.10)	3.59 (1.19)	3.44 (1.50)
	... the cost of living	3.64 (1.16)	3.53 (1.33)	3.2 (1.61)
	... protecting the environment	3.59 (1.02)	3.65 (1.20)	3.4 (1.51)
**Familiarity of AI**				
**Importance of information about AI** from	... General media	3.07 (1.16)	2.82 (0.98)	2.77 (1.10)
	... Service providers	2.94 (1.21)	2.51 (1.07)	2.27 (1.11)
	... Word of mouth	3.02 (1.21)	2.73 (1.11)	2.59 (1.18)
	... Social media	2.86 (1.28)	2.61 (1.15)	2.33 (1.16)
	... Social norms	3.12 (1.16)	2.82 (1.08)	2.64 (1.15)
**Proactive interactions with AI**	Recognise AI	2.73 (1.33)	3.18 (1.23)	3.57 (1.34)
	Information seeking on AI	2.76 (1.27)	2.28 (1.05)	2.02 (1.02)
	Frequency of generative AI use	%	%	%
	(Never)	21.9	33.0	52.6
	(Less than monthly)	30.5	44.1	37.2
	(More than monthly)	47.6	23.0	10.1

Supplementary Table 6. a) Descriptive statistics for survey questions on a) AI perceptions (n=1981), b) data privacy concerns (n=2078), and c) usage of four climate-relevant AI applications (n=2078). For data privacy concerns 1=not at all concerned, 5= extremely concerned. For change in data privacy concerns over the past year, >0 indicates greater concern than a year ago.

a)

	Survey items	Current (% of sample)
**AI risks**	More risks than benefits	36
	More benefits than risks	18
	Equal benefits and risks	46
**Positive impacts of AI**	Improve healthcare	36
	Shopping experiences	35
	Access to learning or education	32
	Make job easier*	36
	No positive impacts	28
**Negative impacts of AI**	Use of personal data without consent	72
	Difficult to identify fake news or misinformation	68
	Increased chance of experiencing cybercrime	51
	Put job at risk*	35

*Amongst respondents in employment.

b)

Data privacy concerns	Current mean (SD)	Change over past year mean (SD)
your information …is shared with websites or companies which you don't use	2.85 (1.23)	0.20 (0.62)
your internet usage information is shared with websites or companies which you don't use	3.04 (1.27)	0.25 (0.63)
your personal information is not handled in a legitimate way	3.47 (1.28)	0.34 (0.63)
your private online conversations are being monitored	3.28 (1.44)	0.27 (0.65)
your internet usage is monitored by authorities	2.95 (1.44)	0.19 (0.61)
your personal data is being used to sell you goods or services	3.27 (1.32)	0.26 (0.64)
Data privacy concerns (sum)	18.87 (6.30)	1.52 (2.93)

c)

	% of total sample, n=2078				
**Usage of climate-relevant AI application**	**Current users**	**Discontinued in past year**	**Decrease in use**	**No change in use**	**Increase in use**
**P2P retail**	51.0	11.6	21.1	21.6	8.4
**Bike share scheme**	8.0	5.7	4.1	2.2	1.6
**Food waste reduction app**	17.3	7.3	6.8	5.2	5.3
**Smart thermostat**	29.1	4.1	9.2	13.4	6.5

Supplementary Table 7. Spearman's rank correlation coefficients for variables in Study 2.

	Variable 1	Variable 2	n	Coefficient rho	P (2 tailed)
a)	Perceived AI risks	Current data privacy concerns	1981	0.163	**<0.001**
	Perceived AI risks	Δ Data privacy concerns	1981	0.201	**<0.001**
b)	Δ Data privacy concerns	Δ Usage of P2P retail platform	1301	0.113	**<0.001**
	Δ Data privacy concerns	Δ Usage of bike share scheme	285	0.202	**<0.001**
	Δ Data privacy concerns	Δ Usage of food waste reduction app	512	0.285	**<0.001**
	Δ Data privacy concerns	Δ Usage of smart thermostat	691	0.380	**<0.001**
c)	**Perceived importance of AI info sources**				
	General media (e.g., TV, radio, newspapers)	Perceived AI risks	1689	-0.054	**0.028**
	Service providers/ other specialist organisations	Perceived AI risks	1485	-0.152	**<0.001**
	Talking with friends, family or colleagues	Perceived AI risks	1607	-0.092	**<0.001**
	Social media	Perceived AI risks	1501	-0.130	**<0.001**
	Being aware of what people in general are doing	Perceived AI risks	1646	-0.0.89	**<0.001**
d)	**Proactive interactions with AI**				
	Seeking AI-related information	Perceived AI risks	1981	-0.211	**<0.001**
	Recognising AI	Perceived AI risks	1981	-0.201	**<0.001**
	ChatGPT usage	Perceived AI risks	1981	-0.317	**<0.001**

Δ = change in

Supplementary Table 8. Differences in current data privacy concerns between respondents who currently use an application to those who do not. Mann-Whitney U test results.

		**Non-user current data privacy concerns**		**User current data privacy concerns**				
**Domain**	**Application**	**n**	**mean rank score**	**n**	**mean rank score**	**U**	**z**	**P (2-tailed)**
Retail	P2P retail platform	1018	1028.44	1060	1050.13	550802.500	0.825	0.410
Mobility	Bike share scheme	1911	1044.06	167	987.32	150855.000	-1.173	0.241
Food	Food waste reduction app	1718	1042.38	360	1025.73	304284.500	-0.479	0.632
Home	Smart thermostat	1473	1034.75	605	1051.08	452586.000	0.564	0.573

Supplementary Table 9. Chi squared test results on exposure to AI information sources and perceived AI risks, n=1981.

Sources of information on AI (not exposed/exposed)	Dependent variable	χ^2	df	P (2 tailed)	Cramer's V
General media (e.g., TV, radio, newspapers, websites)	Perceived AI risks	9.259	2	**0.010**	**0.068**
Service providers or other specialist organisations		39.585	2	**< 0.001**	**0.141**
Talking with friends, family or colleagues		16.527	2	**< 0.001**	**0.091**
Social media		28.664	2	**< 0.001**	**0.120**
Being aware of what people in general are doing		24.932	2	**< 0.001**	**0.112**

Supplementary Table 10. Test for mediation of protecting personal data (four climate-relevant AI applications) structural equation model fit indices. Regression imputation was used to deal with missing values for each model to enable bootstrapping, n=2078.

**Pathway** Perceived AI risks → Privacy concern → *Data protection* → [Application] usage frequency	**Model fit**						
	**χ2**	**p**	**d.f.**	**RMSEA**	**CFI**	**TFI**	**AIC**
**Recommended value**				<0.08	>0.95	>0.95	
P2P retail platform	7.290	0.026	2	0.036	0.943	0.829	31.290
Bike share scheme	70.687	<0.001	2	0.129	0.592	-0.225	94.687
Food waste reduction app	28.106	<0.001	2	0.079	0.773	0.319	52.106
Smart thermostat	49.537	<0.001	2	0.107	0.675	0.024	73.537

Supplementary Table 11. Test for mediation of protecting personal data using a bootstrap analysis with a 95% Confidence Interval. Unstandardised coefficients reported. Bootstrap Sample = 5000 with replacement.

**Pathway**	**Perceived AI risks → Data privacy concerns**		**Data privacy concerns → *Data protection*→ [Application] usage frequency**						
							**95% CI**		
**Application**	**Direct effect β**	**P (2-tailed)**	**Direct effect β**	**P (2-tailed)**	**Indirect effect a x b = β**	**P (2-tailed)**	**Low**	**High**	**Mediation conclusion**
P2P retail platform	1.505	**<0.001**	0.000	0.982	0.003	**<0.001**	0.001	0.004	Full
Bike share scheme			-0.010	**0.010**	0.002	**<0.001**	0.001	0.003	Partial
Food waste reduction app			-0.006	0.260	0.002	**0.001**	0.001	0.003	Full
Smart thermostat			-0.001	0.840	0.003	**<0.001**	0.002	0.006	Full

a: Effect of the independent variable on the mediator.
b: Effect of the mediator on the dependent variable.

Supplementary Table 12. Test for mediation of protecting personal data using a bootstrap analysis with a 95% Confidence Interval. Unstandardised coefficients reported. Bootstrap Sample = 5000 with replacement. Grey text indicates the four applications previously investigated.

	**Pathway**	**Perceived AI Risks → Data privacy concerns**		**Data privacy concerns → *Data protection* → [Application] usage frequency**						
**Domain**	**Application**	**Direct effect β**	**P (2-tailed)**	**Direct effect β**	**P (2-tailed)**	**Indirect effect a x b = β**	**P (2-tailed)**	**95% CI** Low	**95% CI** High	**Mediation conclusion**
Retail	Online large retailers			0.006	0.250	0.002	**0.002**	0.001	0.003	Full
	P2P retail platform			0.000	0.982	0.003	**<0.001**	0.001	0.004	Full
	Online small retailers			0.000	0.968	0.004	**<0.001**	0.002	0.006	Full
Mobility	Car clubs			-0.003	0.327	0.001	**<0.001**	0.001	0.002	Full
	P2P car sharing			-0.004	0.252	0.001	**<0.001**	0.001	0.002	Full
	Ride sharing			-0.001	0.805	0.003	**<0.001**	0.001	0.004	Full
	Bike share scheme			-0.010	**0.010**	0.002	**<0.001**	0.001	0.003	Partial
Food	Online grocery shopping	1.505	**<0.001**	-0.006	0.375	0.003	**<0.001**	0.001	0.005	Full
	Online meal delivery			0.000	0.974	0.004	**<0.001**	0.002	0.006	Full
	Meal kits			-0.009	**0.048**	0.002	**<0.001**	0.001	0.003	Full
	Food waste reduction app			-0.006	0.260	0.002	**0.001**	0.001	0.003	Full
Home	Smart thermostat			-0.001	0.840	0.003	**<0.001**	0.002	0.006	Full
	Learn about energy use			0.011	0.112	0.006	**<0.001**	0.003	0.009	Full
	Learn how to reduce energy use			0.025	**<0.001**	0.006	**<0.001**	0.003	0.009	Partial
	Shift time of energy use			-0.007	0.352	0.004	**<0.001**	0.002	0.007	Full

a: Effect of the independent variable on the mediator.
b: Effect of the mediator on the dependent variab

Supplementary Table 13. Test for mediation of protecting personal data (15 climate-relevant AI applications) structural equation model fit indices. Grey text indicates the four applications previously investigated. Regression imputation was used to deal with missing values to enable bootstrapping, n=2078.

**Pathway -** Perceived AI risks → Privacy concern → *Data protection* → [Application] usage frequency		**Model fit** χ2	**P**	**d.f.**	**RMSEA**	**CFI**	**TFI**	**AIC**
	**Recommended value**				<0.08	>0.95	>0.95	
**Domain**	**Application**							
**Retail**	Online large retailers	24.182	<0.001	2	0.073	0.799	0.397	48.182
	P2P retail platform	7.290	0.026	2	0.036	0.943	0.829	31.290
	Online small retailers	12.542	0.002	2	0.050	0.911	0.732	36.542
**Mobility**	Car clubs	66.876	<0.001	2	0.125	0.578	-0.266	90.876
	P2P car sharing	56.247	<0.001	2	0.114	0.621	-0.138	80.247
	Ride sharing	76.625	<0.001	2	0.134	0.575	-0.274	100.625
	Bike share scheme	70.687	<0.001	2	0.129	0.592	-0.225	94.687
**Food**	Online grocery shopping	26.813	<0.001	2	0.077	0.787	0.360	50.813
	Online meal delivery	52.471	<0.001	2	0.110	0.675	0.024	76.471
	Meal kits	68.091	<0.001	2	0.126	0.587	-0.238	92.091
	Food waste reduction app	28.106	<0.001	2	0.079	0.773	0.319	52.106
**Home**	Smart thermostat	49.537	<0.001	2	0.107	0.675	0.024	73.537
	Learn about energy use	20.177	<0.001	2	0.066	0.891	0.672	44.177
	Learn how to reduce energy use	16.792	<0.001	2	0.060	0.917	0.750	40.792
	Shift time of energy use	17.314	<0.001	2	0.061	0.881	0.642	41.314

Supplementary Table 14. Socio demographics of survey sample, n=2078.

Respondent characteristics		Percentage of survey sample %
**Gender**	Male	48.9
	Female	50.9
**Age**	18-24	13.6
	25-34	15.1
	35-44	15.4
	45-54	14.5
	55-64	14.5
	65-74	13.5
	75+	13.5
**Education**	Secondary or lower	8.7
	College or other	47.0
	Undergraduate or higher	43.3
**Financial situation**	Very difficult	8.2
	Difficult	19.5
	Coping	48.1
	Living comfortably	22.8

Supplementary Table 15. Pilot study results on perceptions of data collection practices and levels of concern about data collection, n=51.

Application	% of sample who perceived [application] collects personal data	Level of concern regarding data collection practices*
P2P retail platform	90.20%	High
Bike share scheme	86.27%	Low
Food waste reduction app	56.86%	Low
Smart thermostat	60.78%	Medium

*asked only to participants who perceived [application] collects personal data.

Supplementary Table 16. Respondent characteristics for the eight vignette sub-samples and statistical test results for differences between sub-samples (survey respondents were randomly assigned to a vignette).

		Retail		Mobility		Food		Home		Statistical significance between sub-groups	
		AI_high (n=250)	AI_low (n=246)	AI_high (n=275)	AI_low (n=245)	AI_high (n=264)	AI_low (n=262)	AI_high (n=267)	AI_low (n=268)		
**Respondent characteristics**		%	%	%	%	%	%	%	%	n	Test result
**Gender**	Male	50	47.2	45.1	47.8	54.3	49.2	47.9	49.6	2073	χ2(7) = 5.725, p = 0.572.
	Female	49.6	52.8	54.9	52.2	45.3	50	51.7	50.4		
**Age**	18-24	14	13.4	13.8	12.7	14.3	14.9	13.9	11.9	2078	χ2(7) = 7.286, p = 0.400.
	25-34	10.8	14.6	15.3	15.9	17.4	13.4	17.2	15.7		
	35-44	15.2	13	16.7	14.3	14	15.3	16.5	17.5		
	45-54	11.6	17.5	14.9	14.7	13.2	15.3	15.4	13.8		
	55-64	19.2	11	15.3	15.1	13.2	13	14.6	14.6		
	65-74	12.4	13.4	13.8	10.6	16.6	13.7	13.5	13.4		
	75+	16.8	17.1	10.2	16.7	11.3	14.5	9	13.1		
**Education**	Secondary or lower	7.2	9.3	6.2	9	9.1	6.1	12.4	10.1	2057	χ2(14) = 16.394, p = 0.290.
	College or other	47.6	46.3	51.3	50.2	46.4	50.4	42.3	41.8		
	Undergraduate or higher	44.4	42.3	41.5	40.4	44.2	42.7	43.8	47		
**Financial situation**	Very difficult	8.8	6.9	8	8.6	9.8	7.6	8.6	7.5	2051	χ2(21) = 12.384, p = 0.929.
	Difficult	16.8	22.4	19.6	20	20.4	18.3	21.3	17.5		
	Coping	51.6	45.9	50.5	42.9	45.7	49.2	49.1	49.6		
	Living comfortably	22	22.8	20.7	27.3	22.6	23.3	19.9	24.3		
**Perceived AI risks**	More benefits	17.2	13.4	16	15.5	17	17.9	17.6	20.1	1981	χ2(14) = 13.244, p = 0.507.
	Equal benefits and risks	38.8	50	41.5	42.4	43	43.9	46.8	44		
	More risks	38.4	32.1	38.5	35.9	35.1	34.7	31.5	31		
		Mean (SD)	Mean (SD)	Mean (SD)	Mean (SD)	Mean (SD)	Mean (SD)	Mean (SD)	Mean (SD)		
	Technophilia score (max.30)	18.20 (4.19)	18.63 (3.85)	17.99 (4.26)	18.27 (4.15)	18.52 (4.49)	18.61 (4.37)	18.53 (4.33)	18.34 (4.37)	2078	χ2(7) = 6.370, p = 0.497.
	Current data privacy concerns	18.3 (6.36)	19.05 (6.39)	19.15 (6.35)	19.24 (5.97)	18.69 (6.38)	18.98 (6.25)	18.64 (6.33)	18.92 (6.37)	2078	χ2(7) = 4.690, p = .698.

Supplementary Table 17. Spearman's rank correlation coefficients between data privacy concerns and application usage frequency.

Domain	Variable 1, Data privacy concerns and Variable 2, [application] usage frequency	n	Coefficient rho	P (2 tailed)
Retail	Online large retailers	2077	0.041	0.061
	P2P retail platform	2077	0.009	0.694
	Online small retailers	2078	0.018	0.409
Mobility	Car clubs	2075	-0.009	0.690
	P2P car sharing	2078	-0.01	0.664
	Ride sharing	2076	0.016	0.478
	Bike share scheme	2076	-0.024	0.277
Food	Online grocery shopping	2076	-0.006	0.800
	Online meal delivery	2078	0.020	0.369
	Meal kits	2078	-0.020	0.351
	Food waste reduction app	2076	-0.012	0.582
Home	Smart thermostat	2069	0.011	0.610
	Learn about energy use	2077	0.055	**0.013****
	Learn how to reduce energy use	2073	0.103	**0.001*****
	Shift time of energy use	2077	0.001	0.971
